# Editorial: Neoadjuvant treatment for resectable and borderline resectable pancreatic cancer

**DOI:** 10.3389/fonc.2023.1138587

**Published:** 2023-01-25

**Authors:** Marco Massani, Tommaso Stecca

**Affiliations:** First General Surgery Unit, HPB Referral Center, ULSS2 Marca Trevigiana, Treviso, Italy

**Keywords:** pancreatic cancer, borderline resectable pancreatic cancer (BRPC), locally advanced pancreatic cancer (LAPC), Neoadjuvant chemo(radio)therapy, lymphnode metastasis

Pancreatic ductal adenocarcinoma (PDAC) is a growing source of cancer-related death. According to the National Cancer Institute (Surveillance, Epidemiology, and End Results Program), in 2022 it has been the third leading cause of cancer-related deaths in the USA. Survival rates have not improved in the last few decades and 5-year relative survival is 11.5%. Its high-mortality rate is attributed to pancreatic cancer biology and difficulty in early diagnosis. Approximately 20% of patients have a resectable or a borderline resectable cancer at diagnosis (1). The American Joint Committee on Cancer (AJCC) tumor, node, and metastasis classification is used to assess prognosis (2); resectability is assessed to select treatment for localized PDAC (3).

Each case is defined as resectable, borderline resectable (BRPC), or locally advanced (LAPC) according to the degree of tumor contact and invasion into the superior mesenteric, gastroduodenal, hepatic artery, or portal vein. A multidisciplinary team (surgical oncologist, radiologist, medical oncologist, and radiation oncologist) assess each case to guide the best treatment option. PDAC is considered resectable when there is less than 180 degrees contact with major vessels. BRPC may have venous and/or partial arterial involvement which defines two distinct categories of tumors: tumors which invade the Portal Vein and/or the Superior Mesenteric Vein and tumors which invade major arteries. The prognosis associated with these two categories of tumors and associated proposed surgical treatment differs significantly (4, 5). Locally advanced PDAC is unresectable at presentation due to vessel invasion (6–9). Furthermore, lymph node metastatic involvement, which develops in 60-70% of patients and is one of the most important prognostic factors, is a topic of debate. Tumor regression in lymph node metastases may demonstrate a biologic benefit of neoadjuvant chemotherapy compared to a surgery first approach (10, 11).

Systemic therapy role in patients with resectable and borderline resectable disease has been most studied in the post-operative setting. The PRODIGE-24 trial, published in 2018, compared 6 months of adjuvant mFOLFIRINOX (modified fluorouracil plus leucovorin, oxaliplatin and irinotecan) to gemcitabine, demonstrating an increased disease-free survival (from 12,8 to 21,6 months) and median overall survival (from 35 to 54,4 months) with the combination therapy. These data made possible to recommend 6 months of adjuvant mFOLFIRINOX as a standard of care for patients with a good performance status after resection of pancreatic ductal adenocarcinoma of any stage. Patients with contraindications to mFOLFIRINOX or with suboptimal performance status receive gemcitabine with or without capecitabine as adjuvant therapy (12).

Achieving an R0 resection is the only curative option. Nevertheless, many R0-resected patients will relapse within 2 years from surgery. Neoadjuvant treatment has been explored in order to improve survival. Theoretical advantages of neoadjuvant treatment include increased R0 resection rate, early delivery of systemic therapy to all patients (addressing occult metastatic disease), and improved patient selection for resection. Many single-arm studies have shown the safety of neoadjuvant therapy (13–15). The final results of the phase 3 PREOPANC-1 study has been published in 2020. Although the Authors found no statistically significant difference in survival, neoadjuvant chemoradiation was associated with a higher R0 resection rate and significantly better disease-free survival (16).

Within this Special Issue, the case report by Lu et al. “*Pathologic Complete Response to Induction Therapy in a Patient With Potentially Resectable Pancreatic Cancer*” reports the use of induction tislelizumab (a PD-1 monoclonal antibody) plus chemoradiotherapy (gemcitabine/nab-paclitaxel plus concurrent tomotherapy) and the change of ctDNA during treatment in a patient diagnosed with Borderline Resectable Pancreatic Head Adenocarcinoma. The Authors relate about the pathologic complete response after 4 cycles of combined therapy and show the dynamic changes of ctDNA during treatment from baseline condition.

The work by Choi et al. “*Proper Adjuvant Therapy in Patients with Borderline Resectable and Locally Advanced Pancreatic Cancer Who Had Received Neoadjuvant FOLFIRINOX*” is a retrospective, single center analysis on 144 patients with borderline resectable and locally advanced pancreatic cancer who underwent radical resection after neoadjuvant FOLFIRINOX with/without concurrent radiotherapy. After radical resection, patients received 5-fluorouracil (5-FU)-based or non-5-FU-based adjuvant therapy. Among the patients who received neoadjuvant radiotherapy, patients with 5-FU based adjuvant therapy had a lower risk of recurrence in comparison with patients with non-5-FU-based.

The paper *“Study protocol for a prospective, open-label, single-arm, phase II study on the combination of tislelizumab, nabpaclitaxel, gemcitabine, and concurrent radiotherapy as the induction therapy for patients with locally advanced and borderline resectable pancreatic cancer.*” by Lu et al. proposes a prospective, open-label, single-arm and single-centre phase II study. The study population is composed by patients aged ≥ 18 years with histologically or cytologically and radiographically confirmed LAPC or BRPC. The regimen consists of four cycles of gemcitabine 1000 mg/m2 and nab-paclitaxel 125mg/m2 administered on day 1 and day 8 *via* intravenous infusion, along with tislelizumab 200 mg administered through IV infusion on day 1 every 3 weeks. Concurrent chemoradiotherapy is administered after two cycles of systemic therapy. The proposed trial attempts to evaluate the safety and efficacy of the combination of anti-PD-1 antibody plus chemotherapy and radiotherapy as induction therapy for LAPC and BRPC to provide evidence for the clinical practice of this treatment.

The Article by Wang et al. “*Neoadjuvant Therapy for Pancreatic Ductal Adenocarcinoma: Where Do We Go?*” is a narrative review aiming to summarize the most recent evidences and controversies in the field of neoadjuvant chemotherapy for pancreatic ductal adenocarcinoma. The review is structured into different sections. Neoadjuvant therapy is discussed with relation to different PDAC stages at presentation, namely borderline resectable and locally advanced, and resectable PDAC. Subsequently, immunotherapy and targeted therapy combined with neoadjuvant chemotherapy are discussed. Eventually, the Authors briefly relate upon radiologic reassessment in clinical practice and timing of surgical exploration after NAT.

This Research Topic aims to provide an update on the current state of neoadjuvant treatment in pancreatic cancer. Currently, clinical trials are ongoing to compare neoadjuvant therapy with upfront surgical resection followed by adjuvant therapy. Probably soon this approach will become a standard of care among borderline resectable, and will be unveiled its role in selected resectable patients and patients with suspected lymphnode metastases too.

## Author contributions

Both Authors (Editor MM and co-Editor TS) concepted, drafted, and revised the Editorial equally. All authors contributed to the article and approved the submitted version
